# Improvement in 6-Minute Walking Distance after Supervised Exercise Training Is Related to Changes in Quality of Life in Patients with Lower Extremity Peripheral Artery Disease

**DOI:** 10.3390/jcm10153330

**Published:** 2021-07-28

**Authors:** Stefano Lanzi, Luca Calanca, André Berchtold, Lucia Mazzolai

**Affiliations:** 1 Heart and Vessel Department, Division of Angiology, Lausanne University Hospital, 1011 Lausanne, Switzerland; luca.calanca@chuv.ch (L.C.); lucia.mazzolai@chuv.ch (L.M.); 2 Faculty of Social and Political Sciences, Institute of Social Sciences (ISS) & The National Centre of Competence in Research (NCCR) LIVES, University of Lausanne, 1015 Lausanne, Switzerland; andre.berchtold@unil.ch

**Keywords:** vascular rehabilitation, walking performance, intermittent claudication, exercise therapy

## Abstract

This study aimed to investigate the relationship between supervised exercise training (SET)-induced changes in treadmill performance and 6 min walking distance, and changes in general (physical and mental) self-perceived health-related quality of life (HRQoL) in symptomatic patients with lower extremity peripheral artery disease (PAD). This is an observational study investigating Fontaine stage II PAD patients participating in 3-month SET. Before and following SET, treadmill performance (pain-free (PFWD) and maximal (MWD)), and 6 min walking distance (6MWD) were assessed. Self-perceived HRQoL was assessed with the Medical Outcomes Study Short-Form 36 (SF-36). Ankle- and toe-brachial indexes were also measured. One-hundred forty-seven patients with PAD were included (64.9 ± 9.6 y, 70% men). After SET, PFWD (+102%, *p* ≤ 0.001), MWD (+87%, *p* ≤ 0.001), and 6MWD (+14%, *p* ≤ 0.001) significantly increased. All eight SF-36 subscale scores significantly improved following SET (*p* ≤ 0.04). SET significantly improved physical and mental component summaries of the SF-36 (*p* ≤ 0.001). Larger increases in 6MWD were associated with greater improvements in physical (*β* = 0.19; *p* = 0.02) and mental (*β* = 0.24; *p* = 0.005) component summaries of the SF-36. No significant relationship was observed between changes in treadmill performance and changes in physical and mental component summaries of the SF-36. These results show that improvements in 6MWD following SET are related to improvements in general self-perceived HRQoL in patients with symptomatic lower extremity PAD. On the contrary, changes in treadmill performance were not related to improvements in HRQoL. These results suggest that the 6 min walking test is an essential outcome measure to assess overall patient functional status following interventions in patients with PAD.

## 1. Introduction

Lower extremity peripheral artery disease (PAD) is mostly secondary to atherosclerotic arterial narrowing, resulting in reduced blood flow and oxygen delivery to the lower limbs [1,2]. Patients with PAD have impaired walking performances and faster functional decline compared to healthy age-matched subjects [3,4]. Consequently, these patients reduce their daily life physical activity, adopting a more sedentary lifestyle behavior, leading to further functional decline [5]. These functional limitations reduce health-related quality of life (HRQoL), which reflects a patient’s physical, mental, and social perceptions in relation to health [6].

Supervised exercise training (SET) is among the guideline-recommended therapies for PAD [1,2]. It is well known that SET increases treadmill performance (pain-free (PFWD) and maximal (MWD) walking distance) and 6 min walking distance (6MWD) compared to usual care [7,8,9,10,11]. In addition, although often regarded as a secondary outcome, SET increases self-perceived HRQoL [7,11,12,13]. This latter result is promising as poor HRQoL is associated with higher rates of mortality in patients with PAD [14]. However, the relationship between a patient’s perceived improvements in general (physical and mental) HRQoL and changes in treadmill performance and 6MWD following SET remains to be clearly determined. This has clinical implications since treadmill performance and 6MWD are commonly used outcome measures for assessing SET interventions in patients with claudication. A recent study showed that greater 6MWD independently predicts better physical and social aspects of quality of life in patients with PAD [15]. However, that study had a cross-sectional design and did not assess treadmill performance [15]. In addition, it has been demonstrated that changes in 6MWD following exercise therapy correlate with changes in daily life physical activity and self-perceived walking distance in patients with PAD [16]. Interestingly, no relationship was observed with changes in treadmill performance [16]. A possible explanation is that, compared to the maximal treadmill test, the 6 min walking is a submaximal test that is more representative of daily life walking. As (walking) daily life activities are usually performed at submaximal levels, the 6 min walking test may better represent overall patient functional status and better relate to a patient’s perceptions in relation to health [16,17].

The present study aims to evaluate the relationship between SET-induced changes in treadmill performance and 6 min walking distance, and changes in general (physical and mental) self-perceived HRQoL in patients with symptomatic PAD. We hypothesized that changes in 6MWD are related to changes in self-perceived HRQoL. On the contrary, we expected no relationship between changes in treadmill performance and improvements in HRQoL.

## 2. Methods

### 2.1. Study Population 

Symptomatic patients with chronic PAD included in the Angiofit study at the Division of Angiology of the University Hospital of Lausanne, Switzerland, were investigated. The Angiofit study is a longitudinal observational cohort of symptomatic patients (Fontaine stage II) participating in multimodal SET with stable uni- or bilateral lower limb claudication and resting ankle-brachial index (ABI) ≤ 0.9 or decreased post-exercise ABI > 20%. We recently showed that our clinical multimodal SET is effective in improving walking performance and quality of life [18,19,20] and that these improvements are maintained up to 12 months follow-up in patients with symptomatic PAD [20]. Compared to these previous observations [18,19,20], in the present study we included all patients participating in our clinical SET program up to 2020, having performed walking tests (treadmill and 6 min) and self-perceived HRQoL questionnaire. Exclusion criteria are inability to participate in SET three times weekly, critical limb ischemia, and cardiac contraindication to exercise. The Angiofit study was approved by the local ethics committee and was conducted according to the Declaration of Helsinki. Subjects provided written, voluntary, informed consent.

### 2.2. Multimodal Supervised Exercise Training Program

The clinical multimodal SET program was coordinated by an angiologist and directly supervised by an exercise physiologist (SL), as recently described elsewhere [18,19,20]. SET consisted of 36 sessions lasting 30–60 min, spread over a 3-month period (3x/week). Twice weekly, patients performed outdoor Nordic walking, and once weekly indoor circuit training focused on strengthening of lower limbs [18,19,20]. During Nordic walking sessions, patients were encouraged to exercise until development of moderate-severe claudication before resting [11]. Patients started again to exercise once pain disappeared. Exercise intensity was mainly set at moderate intensity (12–14 on Borg’s scale) [21]. The indoor circuit training included specific functional walking (toe/heel, high knees, side-to-side, backward walking) or bodyweight resistance exercises (squat, calf/heel raise, lunges) focused on the main lower limb muscles. At the beginning of SET, patients were asked to perform 5–15 repetitions of each bodyweight resistance exercise interspersed with 30 to 60 s of recovery and were encouraged to exercise at low intensity (9–11 on Borg’s scale). Subsequently, if tolerated, patients were encouraged to exercise at moderate intensity (12–14 on Borg’s scale). To that end, patients were encouraged to perform bodyweight resistance exercises (e.g., 20–30 repetitions) or 10–20 repetitions using dumbbells or elastic bands. Each training session started with a 5 min warm-up focused on coordination and balance and ended with a 5 min cool-down period. During SET, patients participated in a 6 h structured therapeutic education workshop on PAD, tobacco, nutrition, and physical activity. The attendance rate to SET was defined as the percentage of attended sessions out of the total number of sessions.

### 2.3. Clinical and Functional Assessments

All assessments were routinely performed prior and following SET.

### 2.4. Patients’ Physical Characteristics 

Physical examination, personal and medical history, and concomitant medications were assessed. Body mass index (BMI), resting heart rate (HR), systolic (SBP) and diastolic (DBP) blood pressure were recorded.

### 2.5. Vascular Parameters

ABI and toe-brachial indexes (TBI) were measured in supine position, as described elsewhere [18,19,20]. Briefly, ABI was measured using a hand-held 5 MHz Doppler flow meter (Atys Medical, Soucieu-en-Jarrest, France), and calculated as the ratio of ankle (*dorsalis pedis* and *posterior tibial*) systolic blood pressure of each limb to the highest brachial SBP. ABI post-exercise was also measured [1]. TBI was calculated using photoplethysmography (Atys Medical, Soucieu-en-Jarrest, France) as the ratio of toe pressure to the highest brachial SBP. The highest ABI and TBI values of the most symptomatic leg were considered.

### 2.6. Constant-Load Treadmill Test

To determine PFWD and MWD, patients performed a constant-load treadmill test which was performed at 3.2 km/h with a 12% slope [11]. Speed was adapted depending on patients’ safety and exercise tolerance ensuring that the same parameters were used before and following SET.

### 2.7. Six-Minute Walking Test

Patients were asked to walk as far as possible within 6 min in an indoor 50 m corridor to determine the 6MWD. Patients were allowed to stop during the test. While resting, they were allowed to lean against the wall, but they were instructed to resume walking as soon as they could, according to guidelines [22].

### 2.8. Health-Related Quality of Life

Medical Outcomes Study Short-Form 36 (SF-36) was used to evaluate self-perceived HRQoL [23]. SF-36 is a validated generic self-administrated questionnaire assessing physical and mental components of health in eight subscales scored on a 0–100 scale (0 (worse score) to 100 (best score)) [23]. Physical component (PCS) and mental component (MCS) summaries were computed using weighting coefficients of a representative sample of people living in Switzerland [24]. PCS is a weighted composite score of physical functioning, physical role functioning, bodily pain, and general health subscales. MCS is a weighted composite score of vitality, social role functioning, emotional role functioning, and mental health [23,24]. 

### 2.9. Statistical Analysis

An intention-to-treat analysis approach was used. First, multiple imputations were performed for patients who failed to complete the SET program [25,26]. T-tests and chi-square tests were used to compare baseline characteristics of patients who completed vs. those that failed to complete the SET program. Multiple imputations for missing data were performed to obtain twenty imputed data sets. We used a fully conditional specification with predictive mean matching to impute all variables simultaneously [25,26]. Age, sex, BMI, cardiovascular risks factors, comorbidities, ongoing treatment, vascular parameters, and baseline outcome values were used to impute the data sets. Each individual item of the SF-36 questionnaire was imputed, and summary scores were computed from the imputed variables. Statistical (Kolmogorov–Smirnov test) and visual assessments of the normality of distribution were performed. Comparisons between before and after SET program were assessed using generalized linear model adjusted for covariates (age, sex), when appropriate. In order to assess whether changes in treadmill performance (delta PFWD and MWD) and 6 min walking distance (delta 6MWD) were related to changes in self-perceived HRQoL (delta SF-36 scores) following SET, simple and multiple regression models were computed. Covariates were added to the models when appropriate. Multicollinearity was assessed to confirm that the independent variables were not highly correlated to each other. This assumption was tested using variance inflation factor values. Statistical analyses were performed with SPSS 27 software (IBM Corporation, Armonk, NY, USA). Level of significance was set at *p* ≤ 0.05.

## 3. Results

### 3.1. Study Population

One hundred forty-seven symptomatic patients with chronic PAD were included (Table 1). Of these, one hundred twenty-nine (88%) completed the SET program. Reasons for not completing SET include dropping out for endovascular revascularization due to worsening of claudication symptoms during SET (*n* = 4), and other reasons (such as health reasons not related to SET, and work commitments, *n* = 14). Baseline characteristics (age, sex, cardiovascular risks factors, vascular parameters, and baseline outcome values) of patients who completed SET were similar to those who failed to complete SET. The attendance rate to SET was 87 ± 2%.

### 3.2. Treadmill Performance and 6 Min Walking Distance

PFWD (+102%), MWD (+87%), and 6MWD (+14%) significantly increased after SET (Table 2). 

### 3.3. Self-Perceived Health-Related Quality of Life

The eight subscale scores of the SF-36 significantly increased (best scores) after SET (Table 3). Similarly, physical and mental component summaries of the SF-36 significantly improved after SET (Table 3). 

### 3.4. Changes in Treadmill Performance and 6 Min Walking Distance and Their Relationship to Changes in Self-Perceived Health-Related Quality of Life Following SET

Larger increases in 6MWD were associated with greater improvements in physical functioning, bodily pain, general health, vitality, and emotional role functioning subscale scores of the SF-36 (Table 4). No significant relationship was observed between changes in 6MWD and changes in physical role functioning, social role functioning, and mental health subscale scores of the SF-36 (Table 4). Larger increases in 6MWD were associated with greater improvements in physical and mental component summaries of the SF-36 (Table 4). No significant relationship was observed between changes in treadmill performance and changes in SF-36 subscales and summary components (Table 4). These results did not substantially change when analyses were performed without multiple imputations (Table 5). 

### 3.5. Vascular Parameters and Physical Characteristics

ABI at rest and after treadmill test remained unchanged after SET (Table 6). TBI significantly increased after SET (mean difference: 0.03) (Table 6). Resting HR, SBP, DBP, body mass, and BMI, were unchanged after SET (Table 6).

## 4. Discussion

Results presented herein confirm our hypothesis and show that larger increases in 6MWD were associated with greater improvements in physical and mental self-perceived HRQoL in patients with symptomatic PAD. On the contrary, changes in treadmill performance were not related to improvements in HRQoL. 

The association between patients’ perceived improvements in quality of life and changes in treadmill (PFWD and MWD) and functional (6MWD) walking performance following exercise therapy is understudied in patients with symptomatic PAD. From a clinical standpoint, this has a great importance since treadmill performance and 6MWD are commonly used outcome measures for assessing SET interventions in patients with claudication. The novelty of the present investigation is that larger increases in 6MWD following SET were associated with greater improvements in physical and mental self-perceived HRQoL. On the contrary, changes in treadmill performance were not related to improvements in HRQoL. These results suggest the importance of changes in functional walking on improvements in HRQoL. These results are in line with recent findings showing that changes in 6MWD following 6-month exercise therapy correlate with changes in daily life physical activity and self-perceived walking distance in patients with PAD [16]. It is interesting to note that the significant correlation between changes in 6MWD and daily life physical activity was reported following different types of exercise interventions [16]. Indeed, this correlation was reported following a home-based overground walking training in the GOALS trial and following supervised treadmill training in the SILC trial as well [16]. In contrast, no relationship was observed with changes in treadmill performance [16]. We speculate that the results of the present investigation are linked to the more functional characteristics of the 6 min walking test. Although the treadmill test is widely performed to determine maximal exercise capacity, this test is not representative of daily life walking conditions [16,17]. Indeed, patients have to maintain a specific walking speed on an inclined (incremental or constant [11]) treadmill slope and usually hold the treadmill handrail to maintain balance [17]. On the other hand, the 6 min walking is a submaximal test that is performed on a flat corridor where patients can choose the walking speed and are allowed to stop and rest. As (walking) daily life activities are usually performed at submaximal levels, the 6 min walking test may better represent functional exertion in these conditions. Taken together, our results suggest that the 6 min walking test is an essential outcome measure to assess overall patient functional status following interventions [16,17]. Our results are also in line with recent findings showing that SF-36 scores are mainly predicted by physical functional markers such as 6MWD, history of stumbling, and daily walking cadence in patients with PAD [27]. In addition, Golledge et al. [15] recently showed that 6MWD independently predicts physical and social aspects of quality of life in patients with PAD. These results suggest that interventions aiming at improving functional (walking) daily life activities are expected to have greater improvements in self-perceived HRQoL [27]. Further studies are needed to better assess this speculation in the future. 

Self-perceived HRQoL following exercise therapy have not been widely investigated in patients with PAD, and the findings are inconsistent [7,12,13]. Indeed, it has been previously shown that 3- [28,29,30], or 6/12-month [31,32,33] exercise therapy improves a patient’s perception of physical HRQoL, with less (or no) effect on mental HRQoL. On the contrary, other studies showed no change in self-perceived physical and mental HRQoL following exercise therapies of similar durations [34,35,36]. Reasons for discrepancies among studies may be related to differences in study population (age, comorbidities, medical history, baseline overall physical function), and/or in exercise training modalities. Results presented herein showed that 3-month multimodal SET improves patients’ overall perceptions of physical and mental HRQoL. Although the design of the present study cannot discriminate whether multimodal SET is superior to treadmill and/or other training modalities, we speculate that improvements in overall self-perceived HRQoL may be related to functional characteristics of our multimodal SET. More research is needed to investigate the effects of different training modalities in improving self-perceived HRQoL in patients with symptomatic PAD. 

Some methodological limitations need to be addressed. First, exercise training was 3 months in duration. If these results are also applicable to traditional longer (6 months) or shorter training durations remains to be determined. Second, missing data had to be replaced using multiple imputations. However, this procedure allowed increasing statistical power and avoided possible selection bias. In addition, the results did not substantially change when analyses were performed without multiple imputations, highlighting the robustness of our imputation method. Third, the treadmill protocol used to evaluate walking capacities may have influenced the treadmill outcomes. It has been demonstrated that the type of intervention (home-based overground walking vs. treadmill training) may result in difference in outcome in patients with PAD [16]. Indeed, treadmill training results in improved treadmill performance but not (or to a lesser extent) in 6MWD, whereas overground walking (common in home-based programs) elicits greater improvements in 6MWD and fewer changes in treadmill performance [16]. It is, however, interesting to note that in the present investigation, where patients performed an overground functional exercise therapy program, treadmill performance improved more than the 6MWD. This may be related to the treadmill protocol. Indeed, constant-load treadmill protocols have lower test–retest reliability and have a more pronounced learning/placebo effect than graded protocols [37]. However, in the present study we used a constant-load protocol with a 12% slope, which is the grade with the greatest test–retest reliability when using constant-load protocols [38]. Fourth, we used a generic questionnaire of quality of life (SF-36) and it would be interesting in the future to also assess associations of treadmill and functional walking performance with disease-specific quality of life questionnaires, such as the Walking Impairment Questionnaire (WIQ), the Peripheral Arterial Occlusive Disease 86 (PAVK-86) questionnaire, the Intermittent Claudication questionnaire, or the VascuQoL questionnaire. 

In conclusion, the results presented herein showed that improvements in 6MWD following SET are related to improvements in general self-perceived HRQoL in patients with symptomatic PAD. On the contrary, changes in treadmill performance were not related to improvements in HRQoL. These results suggest that the 6 min walking test is an essential outcome measure to assess overall patient functional status following interventions.

## Figures and Tables

**Table 1:** Baseline characteristics of the study population.

Characteristics	Mean ± SD or *n* (%)
Number of included patients	147
Age—y	64.9 ± 9.6
BMI—kg·m^−2^	27.6 ± 5.1
Women	44 (30)
Cardiovascular risks factors	
Type 2 diabetes mellitus	51 (35)
Hypertension	105 (71)
Smoking (current)	74 (50)
Smoking (former)	58 (40)
Smoking (never)	15 (10)
Hypercholesterolemia	117 (80)
Family history of CVD	37 (25)
Prior history of CVD	
Cardiac	43 (29)
Cerebrovascular	11 (7)
Stenosis localization	
Aorto-iliac	25 (17)
Ilio-femoral	12 (8)
Femoro-popliteal	92 (63)
Distal	7 (5)
Proximal-distal	11 (7)
Prior arterial revascularization	81 (55)
Ongoing treatment	
Antiplatelet	136 (93)
Antihypertensive	82 (56)
Lipid lowering	108 (73)
Anticoagulant	15 (10)
Antidiabetic	50 (34)

BMI = body mass index; CVD = cardiovascular disease.

**Table 2:** Treadmill performance and 6 min walking distance before and after supervised exercise training.

Variable	Before	After	%Change	Wald Chi-2	*p* Value
PFWD—m ^a^	105.6 ± 110.8	213.4 ± 231.0	102	21.4	≤0.001
MWD—m	324.1 ± 265.8	606.3 ± 456.5	87	63.3	≤0.001
6MWD—m ^a^	414.9 ± 87.5	471.8 ± 86.4	14	55.6	≤0.001

Values are means ± SD. PFWD = pain-free walking distance; MWD = maximal walking distance; 6MWD = six-minute walking distance. ^a^ adjusted for sex. The influence of covariates (age and sex) on the different outcomes was assessed a priori with simple linear regression. Changes in PFWD (β = 0.17; *p* = 0.04) and 6MWD (β = 0.20; *p* = 0.04) after training were significantly higher in women. In contrast, changes in MWD after training were not related to sex (β = 0.14; *p* = 0.11). Therefore, generalized linear models were adjusted for sex for PFWD and 6MWD. Changes in PFWD, MWD, and 6MWD were not related to age.

**Table 3:** Short-Form 36 scores before and after the supervised exercise training program.

Variable	Before	After	Wald Chi-2	*p* Value
Physical Functioning	53.6 ± 18.4	61.2 ± 18.9	26.7	≤0.001
Physical Role Functioning ^a^	47.6 ± 39.5	65.4 ± 35.1	22.6	≤0.001
Bodily Pain	47.5 ± 19.0	56.2 ± 17.3	30.0	≤0.001
General Health	51.5 ± 16.2	53.9 ± 14.1	4.5	0.04
Physical Component Summary ^a^	31.2 ± 9.7	35.9 ± 9.3	28.8	≤0.001
Vitality	46.5 ± 16.7	52.6 ± 14.8	24.8	≤0.001
Social Role Functioning	61.0 ± 24.2	68.4 ± 21.2	15.4	≤0.001
Emotional Role Functioning	54.9 ± 39.7	71.3 ± 33.2	21.0	≤0.001
Mental Health	64.2 ± 17.1	66.9 ± 15.8	5.6	0.02
Mental Component Summary	41.9 ± 11.6	45.7 ± 10.6	18.2	≤0.001

Values are means ± SD. ^a^ adjusted for sex. The influence of covariates (age and sex) on the different outcomes was assessed a priori with simple linear regression. Changes in physical role functioning score (β = 0.21; *p* = 0.01) and physical component score (β = 0.21; *p* = 0.01) after training were significantly higher in women. In contrast, changes in other scores after training were not related to sex. Therefore, generalized linear models were adjusted for sex for physical role functioning score and physical component score. Changes in all scores were not related to age.

**Table 4:** Changes in treadmill performance and 6 min walking distance and their relationship to changes in health-related quality of life following supervised exercise training with multiple imputations.

Changes in Quality of Life SF-36 Scores	Delta PFWD	Delta MWD	Delta 6MWD
Physical Functioning	β = −0.03; *p* = 0.74	β = 0.002; *p* = 0.98	β = 0.25; *p* = 0.003
Physical Role Functioning ^a^	β = −0.05; *p* = 0.61	β = 0.06; *p* = 0.53	β = 0.12; *p* = 0.15
Bodily Pain	β = 0.02; *p* = 0.86	β = −0.03; *p* = 0.78	β = 0.21; *p* = 0.01
General Health	β = −0.10; *p* = 0.30	β = −0.17; *p* = 0.07	β = 0.21; *p* = 0.01
Physical Component Summary ^a^	β = −0.05; *p* = 0.57	β = −0.01; *p* = 0.92	β = 0.19; *p* = 0.02
Vitality	β = −0.18; *p* = 0.06	β = 0.12; *p* = 0.22	β = 0.26; *p* = 0.002
Social Role Functioning	β = −0.09; *p* = 0.39	β = −0.01; *p* = 0.95	β = 0.16; *p* = 0.06
Emotional Role Functioning	β = 0.02; *p* = 0.81	β = −0.07; *p* = 0.45	β = 0.26; *p* = 0.002
Mental Health	β = −0.06; *p* = 0.51	β = 0.14; *p* = 0.14	β = 0.13; *p* = 0.14
Mental Component Summary	β = −0.06; *p* = 0.51	β = 0.02; *p* = 0.87	β = 0.24; *p* = 0.005

PFWD = pain-free walking distance; MWD = maximal walking distance; 6MWD = six-minute walking distance. *β*: standardized coefficient. ^a^ adjusted for sex.

**Table 5:** Changes in treadmill performance and 6 min walking distance and their relationship to changes in health-related quality of life following supervised exercise training without multiple imputations.

Changes in Quality of Life SF-36 Scores	Delta PFWD	Delta MWD	Delta 6MWD
Physical Functioning	β = −0.07; *p* = 0.50	β = −0.04; *p* = 0.69	β = 0.28; *p* = 0.006
Physical Role Functioning ^a^	β = −0.06; *p* = 0.55	β = 0.08; *p* = 0.45	β = 0.10; *p* = 0.31
Bodily Pain	β = −0.03; *p* = 0.78	β = −0.03; *p* = 0.76	β = 0.26; *p* = 0.009
General Health	β = −0.03; *p* = 0.78	β = −0.14; *p* = 0.18	β = 0.03; *p* = 0.78
Physical Component Summary ^a^	β = −0.07; *p* = 0.51	β = −0.03; *p* = 0.81	β = 0.15; *p* = 0.14
Vitality	β = −0.19; *p* = 0.08	β = 0.20; *p* = 0.06	β = 0.09; *p* = 0.36
Social Role Functioning ^b^	β = −0.08; *p* = 0.46	β = −0.06; *p* = 0.96	β = 0.13; *p* = 0.19
Emotional Role Functioning	β = −0.01; *p* = 0.89	β = −0.06; *p* = 0.55	β = 0.31; *p* = 0.002
Mental Health	β = −0.12; *p* = 0.24	β = 0.16; *p* = 0.13	β = 0.22; *p* = 0.03
Mental Component Summary	β = −0.09; *p* = 0.38	β = 0.02; *p* = 0.86	β = 0.27; *p* = 0.008

PFWD = pain-free walking distance; MWD = maximal walking distance; 6MWD = six-minute walking distance. *β:* standardized coefficient. ^a^ adjusted for sex; ^b^ adjusted for age.

**Table 6:** Vascular parameters and physical characteristics before and after supervised exercise training.

Variable	Before	After	Wald Chi-2	*p* Value
ABI	0.82 ± 0.22	0.81 ± 0.19	0.1	0.73
ABIpEx (% decrement)	43.9 ± 22.6	42.3 ± 21.8	0.8	0.38
TBI	0.60 ± 0.18	0.63 ± 0.16	4.5	0.03
Resting HR—bpm	71 ± 12	69 ± 10	3.5	0.07
SBP—mmHg	145 ± 18	146 ± 18	0.8	0.37
DBP—mmHg	79 ± 11	78 ± 10	0.2	0.67
Body mass—kg	78.1 ± 16.3	78.7 ± 15.8	1.2	0.27
BMI—kg·m^−2^	27.6 ± 5.1	27.9 ± 5.1	1.4	0.24

Values are means ± SD. ABI = ankle-brachial index; ABIpEx = post-exercise ABI; TBI = toe-brachial index; SBP = systolic blood pressure; DBP = diastolic blood pressure; BMI = body mass index. The influence of covariates (age and sex) on the different outcomes was assessed a priori with simple linear regression. Changes in all variables were not related to sex and/or age.

## Data Availability

The data presented in this study are available on request from the corresponding author.

## References

[1] Aboyans V., Ricco J.B., Bartelink M.E.L., Bjorck M., Brodmann M., Cohnert T., Collet J.P., Czerny M., De Carlo M., Naylor A.R. (2018). 2017 ESC Guidelines on the Diagnosis and Treatment of Peripheral Arterial Diseases, in collaboration with the European Society for Vascular Surgery (ESVS): Document covering atherosclerotic disease of extracranial carotid and vertebral, mesenteric, renal, upper and lower extremity arteriesEndorsed by: The European Stroke Organization (ESO)The Task Force for the Diagnosis and Treatment of Peripheral Arterial Diseases of the European Society of Cardiology (ESC) and of the European Society for Vascular Surgery (ESVS). Eur. Heart J..

[2] Frank U., Nikol S., Belch J. (2019). 5 Conservative treatment for PAD—Risk factor management. Vasa.

[3] McDermott M.M., Ferrucci L., Guralnik J.M., Dyer A.R., Liu K., Pearce W.H., Clark E., Liao Y., Criqui M.H. (2010). The ankle-brachial index is associated with the magnitude of impaired walking endurance among men and women with peripheral arterial disease. Vasc. Med..

[4] McDermott M.M., Liu K., Greenland P., Guralnik J.M., Criqui M.H., Chan C., Pearce W.H., Schneider J.R., Ferrucci L., Celic L. (2004). Functional decline in peripheral arterial disease: Associations with the ankle brachial index and leg symptoms. JAMA.

[5] McDermott M.M., Greenland P., Ferrucci L., Criqui M.H., Liu K., Sharma L., Chan C., Celic L., Priyanath A., Guralnik J.M. (2002). Lower extremity performance is associated with daily life physical activity in individuals with and without peripheral arterial disease. J. Am. Geriatr. Soc..

[6] Regensteiner J.G., Hiatt W.R., Coll J.R., Criqui M.H., Treat-Jacobson D., McDermott M.M., Hirsch A.T. (2008). The impact of peripheral arterial disease on health-related quality of life in the Peripheral Arterial Disease Awareness, Risk, and Treatment: New Resources for Survival (PARTNERS) Program. Vasc. Med..

[7] Lane R., Harwood A., Watson L., Leng G.C. (2017). Exercise for intermittent claudication. Cochrane Database Syst. Rev..

[8] Parmenter B.J., Dieberg G., Smart N.A. (2015). Exercise training for management of peripheral arterial disease: A systematic review and meta-analysis. Sports Med..

[9] Parmenter B.J., Mavros Y., Ritti Dias R., King S., Fiatarone Singh M. (2019). Resistance training as a treatment for older persons with peripheral artery disease: A systematic review and meta-analysis. Br. J. Sports Med..

[10] Parmenter B.J., Raymond J., Dinnen P., Singh M.A. (2011). A systematic review of randomized controlled trials: Walking versus alternative exercise prescription as treatment for intermittent claudication. Atherosclerosis.

[11] Treat-Jacobson D., McDermott M.M., Bronas U.G., Campia U., Collins T.C., Criqui M.H., Gardner A.W., Hiatt W.R., Regensteiner J.G., Rich K. (2019). Optimal Exercise Programs for Patients With Peripheral Artery Disease: A Scientific Statement From the American Heart Association. Circulation.

[12] Guidon M., McGee H. (2010). Exercise-based interventions and health-related quality of life in intermittent claudication: A 20-year (1989-2008) review. Eur. J. Cardiovasc. Prev. Rehabil..

[13] Parmenter B.J., Dieberg G., Phipps G., Smart N.A. (2015). Exercise training for health-related quality of life in peripheral artery disease: A systematic review and meta-analysis. Vasc. Med..

[14] Issa S.M., Hoeks S.E., Reimer W.J.S.O., Van Gestel Y.R., Lenzen M.J., Verhagen H.J., Pedersen S.S., Poldermans N. (2010). Health-related quality of life predicts long-term survival in patients with peripheral artery disease. Vasc. Med..

[15] Golledge J., Leicht A.S., Yip L., Rowbotham S.E., Pinchbeck J., Jenkins J.S., Clapperton R., Dally-Watkins M., Singh M.A.F., Mavros Y. (2020). Relationship Between Disease Specific Quality of Life Measures, Physical Performance, and Activity in People with Intermittent Claudication Caused by Peripheral Artery Disease. Eur. J. Vasc. Endovasc. Surg..

[16] McDermott M.M., Guralnik J.M., Tian L., Zhao L., Polonsky T.S., Kibbe M.R., Criqui M.H., Zhang D., Conte M.S., Domanchuk K. (2020). Comparing 6-min walk versus treadmill walking distance as outcomes in randomized trials of peripheral artery disease. J. Vasc. Surg..

[17] McDermott M.M., Guralnik J.M., Criqui M.H., Liu K., Kibbe M.R., Ferrucci L. (2014). Six-minute walk is a better outcome measure than treadmill walking tests in therapeutic trials of patients with peripheral artery disease. Circulation.

[18] Calanca L., Lanzi S., Ney B., Berchtold A., Mazzolai L. (2020). Multimodal Supervised Exercise Significantly Improves Walking Performances Without Changing Hemodynamic Parameters in Patients With Symptomatic Lower Extremity Peripheral Artery Disease. Vasc. Endovasc. Surg..

[19] Lanzi S., Boichat J., Calanca L., Aubertin P., Malatesta D., Mazzolai L. (2021). Gait changes after supervised exercise training in patients with symptomatic lower extremity peripheral artery disease. Vasc. Med..

[20] Ney B., Lanzi S., Calanca L., Mazzolai L. (2021). Multimodal Supervised Exercise Training is Effective in Improving Long Term Walking Performance in Patients with Symptomatic Lower Extremity Peripheral Artery Disease. J. Clin. Med..

[21] Borg G.A. (1982). Psychophysical bases of perceived exertion. Med. Sci. Sports Exerc..

[22] ATS Committee on Proficiency Standards for Clinical Pulmonary Function Laboratories (2002). ATS statement: Guidelines for the six-minute walk test. Am. J. Respir. Crit. Care Med..

[23] Ware J.E., Sherbourne C.D. (1992). The MOS 36-item short-form health survey (SF-36). I. Conceptual framework and item selection. Med. Care.

[24] Roser K., Mader L., Baenziger J., Sommer G., Kuehni C.E., Michel G. (2019). Health-related quality of life in Switzerland: Normative data for the SF-36v2 questionnaire. Qual. Life Res..

[25] Van Buuren S. (2007). Multiple imputation of discrete and continuous data by fully conditional specification. Stat. Methods Med. Res..

[26] Van Buuren S. (2012). Flexible Imputation of Missing Data.

[27] Gardner A.W., Montgomery P.S., Wang M., Xu C. (2018). Predictors of health-related quality of life in patients with symptomatic peripheral artery disease. J. Vasc. Surg..

[28] Gardner A.W., Parker D.E., Montgomery P.S., Scott K.J., Blevins S.M. (2011). Efficacy of quantified home-based exercise and supervised exercise in patients with intermittent claudication: A randomized controlled trial. Circulation.

[29] Patterson R.B., Pinto B., Marcus B., Colucci A., Braun T., Roberts M. (1997). Value of a supervised exercise program for the therapy of arterial claudication. J. Vasc. Surg..

[30] Tsai J.C., Chan P., Wang C.H., Jeng C., Hsieh M.H., Kao P.F., Chen Y.J., Liu J.C. (2002). The effects of exercise training on walking function and perception of health status in elderly patients with peripheral arterial occlusive disease. J. Intern. Med..

[31] Collins E.G., Langbein W.E., Orebaugh C., Bammert C., Hanson K., Reda D., Edwards L.C., Littooy F.N. (2005). Cardiovascular training effect associated with polestriding exercise in patients with peripheral arterial disease. J. Cardiovasc. Nurs..

[32] McDermott M.M., Ades P., Guralnik J.M., Dyer A.R., Ferrucci L., Liu K., Nelson M., Lloyd-Jones D., Van Horn L., Garside D.B. (2009). Treadmill exercise and resistance training in patients with peripheral arterial disease with and without intermittent claudication: A randomized controlled trial. JAMA.

[33] Nicolai S.P., Teijink J.A., Prins M.H. (2010). Exercise Therapy in Peripheral Arterial Disease Study G. Multicenter randomized clinical trial of supervised exercise therapy with or without feedback versus walking advice for intermittent claudication. J. Vasc. Surg..

[34] Guidon M., McGee H. (2013). One-year effect of a supervised exercise programme on functional capacity and quality of life in peripheral arterial disease. Disabil. Rehabil..

[35] Kakkos S.K., Geroulakos G., Nicolaides A.N. (2005). Improvement of the walking ability in intermittent claudication due to superficial femoral artery occlusion with supervised exercise and pneumatic foot and calf compression: A randomised controlled trial. Eur. J. Vasc. Endovasc. Surg..

[36] Savage P., Ricci M.A., Lynn M., Gardner A., Knight S., Brochu M., Ades P. (2001). Effects of home versus supervised exercise for patients with intermittent claudication. J. Cardiopulm. Rehabil..

[37] Hiatt W.R., Rogers R.K., Brass E.P. (2014). The treadmill is a better functional test than the 6-min walk test in therapeutic trials of patients with peripheral artery disease. Circulation.

[38] Nicolaï S.P., Viechtbauer W., Kruidenier L.M., Candel M.J., Prins M.H., Teijink J.A. (2009). Reliability of treadmill testing in peripheral arterial disease: A meta-regression analysis. J. Vasc. Surg..

